# Dataset of thermal behaviour and weather data of thermal disinfestation of *Sitophilus oryzae* in plastic bags using solar heating

**DOI:** 10.1016/j.dib.2022.108029

**Published:** 2022-03-07

**Authors:** Shams Fawki, Ayat Yousery

**Affiliations:** Entomology Department, Faculty of Science, Ain Shams University, Abbasiya, Cairo 11566, Egypt

**Keywords:** Solar flux, Temperature profiles, Heat transfer, Thermal control, Solar disinfestation, Thermal model

## Abstract

This dataset is related to another research paper “Fawki, S., Fields, P.G., Jian, F., Yousery, A., 2022. Control of *Sitophilus oryzae* (Coleoptera: Curculionidae) in bags of wheat using solar radiation. Journal of Stored Products Research 96, 101941. doi:10.1016/j.jspr.2022.101941.” The data was collected in Canada and Egypt. In Canada, Clear polyethylene bags of wheat were used for thermal control using solar radiation. There were four treatments of different wheat amounts, 16, 21, and 25 kg inside clear bags in wood boxes and another 21 kg of wheat in a plastic bag not in a wood box. The solar heating for all treatments was investigated in the field under two different conditions. First, the temperature profile inside the bags was recorded every morning, and the grains were mixed and stacked in foam boxes during the night over five days. Second, the temperature profile was recorded continuously during day and night over six days. Different weather data: ambient temperature, solar radiation, wind direction, and wind speed were collected during both experiments using a weather station located on the field. In Egypt, clear plastic bags and woven plastic bags with 16 kg of wheat were used for solar heating over 5 d. We present the temperature profile data inside the plastic polyethene bags under different storage conditions, grain amounts, bags materials and different weather conditions, which will allow other researchers to develop models to understand the thermal behaviour for thermal disinfestation. Solar heating is a very promising disinfestation technique that was successfully used for museum pest control, postharvest pest control, and soil disinfestation.

## Specifications Table

**Table:** 

Subject	Agricultural Sciences
Specific subject area	Postharvest protection, stored-product pest control, thermal control, food storage.
Type of data	Figures Tables
How data were acquired	In Canada and Egypt, the grain temperatures inside the plastic bags were measured using data loggers (HOBO Thermocouple Data Loggers, Onset Computer Corporation, Bourne, MA, USA) and T-Type thermocouples every 15 min interval. In Canada, the ambient temperature, solar radiation and other environmental data were recorded every 15 min using a Campbell Scientific weather station (Campbell Scientific, Logan, Utah, USA), which was set close to the bags in the field. In Egypt, the weather data was collected on an hourly basis during the experiment from the Egyptian Meteorological Authority, Cairo, Egypt.
Data format	Raw Data. Calculated Data.
Parameters for data collection	In Canada, the grain temperatures inside clear plastic bags were recorded in six positions distributed vertically at the centre of each bag. Temperatures were collected under different conditions of sun exposures, storage system, and grain amounts. There were two main conditions in the field. Mixing grains and stacking the bags experiment (Canada 1) and a second with no-mixing and no-stacking experiment (Canada 2). For both, there were four treatments, 16, 21, 25 kg of wheat inside a plastic bag in a wooden box and another 21 kg of wheat inside a clear bag not in a wooden box. The weather data was also collected for both experiments. These data were ambient air temperature, humidity, air pressure, solar radiation, precipitation, wind speed, and wind direction. In Egypt, there were two different treatments, clear plastic bags and woven plastic bags; each had 16 kg of wheat inside a wood box. The grain temperature profiles were also recorded as mentioned for Canada. The total solar radiation, wind direction and wind speed were collected every hour during solar heating, while the maximum temperature of the day was collected per day.
Description of data collection	Data of grain temperature fluctuations were recorded every 15 min using T-type thermocouples and data loggers. In Canada 1, the grain temperatures were recorded every day during the sun exposure period (from ≈ 10 am to 8 pm) over five days. In Canada 2, the grain temperatures were recorded day and night continuously over six days. During each experiment, the ambient temperature, solar radiation, and other environmental data were recorded every 15 min using a Campbell Scientific weather station, which was set close to the bags in the field. In Egypt, the grain temperature fluctuations were recorded every 15 min using T-type thermocouples and data loggers. The grain temperatures were recorded every day during the sun exposure period (from ≈ 10 am to 3 pm) over five days. Weather data were also collected by the Egyptian Meteorological Authority, Cairo, Egypt (about 1 km away from the experimental location).
Data source location	Canada part: Institution: University of Manitoba. City/Region: Winnipeg, Manitoba. Country: Canada. Data: Grain temperatures and weather data. Egypt part: Institution: Ain Shams University. City: Cairo. Country: Egypt. Data: Grain temperatures and weather data.
Data accessibility	Repository name: Mendeley Data Data identification number (DOI): 10.17632/j9c5mcmw3c.1 Direct URL to data: https://data.mendeley.com/datasets/j9c5mcmw3c/1
Related research article	S. Fawki, P.G. Fields, F. Jian, A. Yousery, Control of *Sitophilus oryzae* (Coleoptera: Curculionidae) in bags of wheat using solar radiation. Journal of Stored Products Research 96 (2022) 101941. doi:10.1016/j.jspr.2022.101941.

## Value of the Data

• The data is useful to understand the thermal behaviour of solar heating for postharvest grain storage.
• Researchers and industry will reuse that data to understand the heat transfer and thermal control for pest disinfestation of grains and other stored products.
• These data are important to develop a thermal model for solar heating using plastic bags/sheets.
• In future, the data will help to improve and compare different materials to be used in solar disinfestation bags.

## 1 Data Description

The dataset represented here is complementary to the data of another research article [1].

All temperature datasets were recorded for solar heating of wheat using clear plastic polyethylene bags. The first part of the dataset was collected in Canada, while the second part was collected in Egypt.

### 1.1 Canadian tests

Grain temperatures inside clear polyethylene bags were recorded in four different treatments: 16, 21, and 25 kg of wheat in plastic bags inside wood boxes and another treatment with 21 kg of wheat in clear bags without a wood box. The grain temperatures of the four treatments were recorded under two different conditions. First, temperatures of solar heating were collected under mixing and stacking conditions over 5 d (Canadian test 1). Second, grain temperatures were collected under no mixing and no stacking condition over 6 d (Canadian test 2). Each replicate in Canadian tests 1 and 2 had six thermocouples to record temperature in six positions at the centre of the plastic bag in the vertical direction. These positions were on the outer surface of the bag (surface), at the top of the grain just beneath the plastic bag (top, inside the bag), between the top and middle of the bag (top-middle), at the middle (middle), between the middle and the bottom (middle-bottom), and at the bottom of the bag (bottom).

#### 1.1.1 Grain bulk with stacking (Canadian test 1)

Grain temperature (°C) fluctuations in clear plastic bags with different amounts of grains as mentioned earlier (Section 1.1) under direct solar radiation every morning for 5 d (Figs. A1–A4 in supplementary figures) [2]. Gaps in the graph between lines correspond to night-time, where the thermocouples were removed, and the bags were stacked in foam boxes.

The weather data collected were temperature (°C), humidity (%), air pressure (kpa), the average Flux density of sun (W/m^2^), total flux of solar radiation (Kj/m^2^), precipitation (MM), wind speed (m/s), and wind direction (°) (Appendices A, Table A.1, supplementary data) [2]. All data were collected every 15 min over 5 d.

The raw data for this experiment is available online (Appendices B, Dataset B1. Canadian test 1 raw data, supplementary data) [2]. These data are represented in an Excel spreadsheet consists of three sheets. The first sheet represents the figures for grain temperatures at the six positions measured in each bag and its corresponding data tables. The second one represents the degree-minutes (DM) (calculation details in Section 2.4) for each treatment over 5 d of the experiment. The last sheet represents the time above 40 °C for each treatment.

#### 1.1.2 Canadian test 2

Grain temperature (°C) fluctuations in clear plastic bags with different amounts of grains as mentioned earlier (Section 1.1) under field conditions day and night over 6 d (Figs. A5–A8 in supplementary figures) [2].

The weather data collected were temperature (°C), humidity (%), air pressure (kpa), the average Flux density of sun (W/m^2^), the total flux of solar radiation (Kj/m^2^), precipitation (MM), wind speed (m/s), and wind direction (°) (Appendices A, Table A.2, supplementary data) [2]. All data were collected every 15 min.

Data is available online in Mendeley Data (Appendices B, Dataset B2. Canada 2 raw data and Dataset B3. Canada 2 raw data, supplementary data) [2]. Dataset B2. Canadian test 2 raw data represents the grain temperatures inside plastic bags for each treatment. Temperatures were recorded every 15 min for 6 d. Dataset B3. Canadian test 2 raw data represents the DM (calculation details in Section 2.4) and time above 40 °C for each treatment.

### 1.2 Egyptian test

Data represents the grain temperatures of 16 kg of wheat in clear plastic bags (Fig. A9 in supplementary figures) [2] and woven plastic bags (Fig. A10 in supplementary figures) [2] over 5 d. The grain was mixed and stacked every day like in Canadian test 1 (Section 1.1.1.).

Weather data were collected over 5 d of the experiment. Wind speed (m/s), wind direction (°), and total solar radiation (Mj/m^2^) were collected every day during the experiment on an hourly basis, while the temperature was collected once per day, the maximum temperature (°C) (Appendices A, Table A3. Egypt, weather data, supplementary data) [2].

Dataset for grain temperatures over 5 d of the experiment is available online (Appendices B, Dataset B4, Egypt raw data) [2]. Three spreadsheets for temperatures inside the plastic bags, time above 40 °C, and DM are also available.

## 2 Experimental Design, Materials and Methods

### 2.1 Grain temperature measurements

Data available represents the temperatures measured for solar heating of wheat inside clear plastic polyethylene bags to control rice weevil *Sitophilus oryzae* (Coleoptera: Curculionidae). Temperatures behaviour inside clear bags were tested under different conditions, different grain amounts, and different bags materials.

In Canada, grain temperatures inside clear bags were recorded in four treatments: 16, 21, and 25 kg of wheat in plastic bags inside wood boxes and another treatment with 21 kg of wheat in clear bags without a wood box. These boxes were open-top square boxes made from 12.5 mm thick plywood (45 cm in length and width). The boxes were made to maintain a uniform shape of the grain bulk at different heights: 9, 13, and 15 cm for 16, 21, and 25 kg of wheat, respectively.

In each bag, the temperatures were recorded in six positions at the centre in a vertical direction. Therefore, six T-Type thermocouples were inserted at the centre of each bag vertically and distributed on equal distances according to different grain bulk heights using heat-shrink tubing. For the 16 kg of wheat, the six thermocouples were located at 0 (outer surface), 0 (inner surface), 2.25, 4.5, 6.75 and 9 cm in the vertical direction to measure temperatures at the following locations: the outer surface of the bag (surface), just beneath the bag's surface from the inner side (top), between top and middle, the middle, between middle and bottom, and the bottom; respectively. For the 21 kg bags, thermocouples were located at 0 (outer surface), 0 (inner surface), 3.25, 6.5, 9.75 and 13 cm depth in the vertical direction to measure temperatures at the six locations mentioned for 16 kg: surface, top of the grain, top-middle, middle, middle-bottom, and bottom. For the 25 kg bags, thermocouples were located at 0 (outer surface), 0 (inner surface), 3.75, 7.5, 11.25 and 15 cm depth in the vertical direction to measure temperatures at the six locations mentioned for 16 kg: surface, top of the grain, top-middle, middle, middle-bottom, and bottom. Each thermocouple set had a square plastic disc attached between the surface and the top thermocouples using an epoxy plastic bonder to keep the position at the centre of the bag. At the beginning of every day, it was important to fix the thermocouples set at the top of the grain bag to ensure that the first thermocouple is outside the bag to measure the surface temperature and the second thermocouple is inside the bag to measure the top temperature.

The grain temperature was measured every 15 min intervals using the HOBO UX120-014M four-channel data loggers and the HOBO UX100-014M a single-channel data loggers (HOBO Thermocouple Data Loggers, Onset Computer Corporation, Bourne, MA, USA).  HOBOware software (version 3.7.16) was used.

### 2.2 Canada

The data was collected in an open field at the University of Manitoba, Winnipeg, Manitoba, Canada. The grain temperatures of the four treatments were recorded under two different conditions. First, temperatures of solar heating were collected under mixing and stacking conditions over 5 d (Canadian test 1). Second, grain temperatures were collected under no mixing and no stacking condition over 6 d (Canadian test 2).

Wheat used was Canadian hard red spring wheat (SY Slate variety, Pitura Seeds) with 12.2 ± 0.0% m.c.. Clear plastic polyethylene bags of 76.2 µm thickness were used (Ploy Bags, Uline, Canada).

All bags in all treatments (12 in total), whether in wood boxes or not, were distributed evenly in a large plywood sheet to uniform the substrate type of the ground.

Weather data and ambient temperature were collected using a Campbell Scientific weather station (Campbell Scientific, Logan, Utah, USA), which was set nearby in the field.

#### 2.2.1 Grain bulk with stacking (Canadian test 1)

Data represent the temperatures of 16, 21, and 25 kg of wheat bags inside boxes and another 21 kg of wheat in the bags without boxes. In order to uniform the grain moisture content (m.c.), the wheat bulk (≈255 kg) was divided into smaller bulks and mixed several times in a rotating metal drum. The initial m.c. of wheat was 12.5 ± 0.0%. The wheat m.c. was measured by using ASABE standard at 130 °C for 19 h [3].

On the day of the sun exposure, all replicates of all treatments (12 in total), whether in wood boxes or not, were distributed evenly in a plywood sheet, 45 cm apart. The plywood layer was used to eliminate any effect from ground type variations.

The grain temperatures were collected from ≈ 11 am to 8 pm for 5 d from 26 to 30 July 2018. At the end of the day, the thermocouples set was removed, and the bag was turned over on the ground several times to mix the grains. Then wheat bags were stacked in the foam box nearby to reduce heat loss during the night. This process was repeated every day.

Bags with 21, 25 kg of wheat in wooden boxes and 21 kg of wheat without a wooden box were stacked in large foam boxes with external dimensions of 87.6 × 110.5 × 81.3 cm. The bag with 16 kg of wheat was stacked in a smaller foam box with external dimensions of 71.1 × 71.1 × 71.1 cm. The foam boxes were made of extruded polystyrene foam Insulation (StyrospanTm, Dow Chemical, Mississauga, Ontario, Canada), and the thickness of the walls of the foam boxes was ≈ 14 cm. The thermal resistance (R) value of the foam is 5.3 m^2^°C/W.

#### 2.2.2 Grain bulk without stacking (Canadian test 2)

The overall design of this part was exactly as previously mentioned (Section 2.2.1.) except that all replicates and all treatments were kept continuously at the field day and night to measure grain temperatures and the effect of solar radiation inside the bags without mixing or stacking of wheat bags. The data were recorded over 6 d from 7 to 12 of August 2018. At night, all replicates were covered with additional wooden boxes to prevent any damage from wild birds and animals in the field.

### 2.3 Egyptian test

Data of grain temperatures of 16 kg of wheat inside plastic bags of different materials were recorded. Wheat was mixed using a shovel to keep a uniform m.c. inside the bag. Clear plastic bags (Makhlouf Group, Cairo, Egypt, 50.8 µm thickness) and woven plastic bags (Makhlouf Group, Cairo, Egypt) were used. Each treatment had three replicates. Experimental design of temperature measurements, mixing of grains, stacking of the bags, and using the foam boxes was exactly as previously mentioned (Sections 2.1., 2.2., and 2.2.1). A plywood sheet (57 × 57 cm) was located under each wood box holding grain bags during the sun exposure. The external dimensions of the foam boxes used were 55 × 55 × 72 cm. These boxes were made from Styrofoam with 13 cm thickness. The bags were subjected to solar radiation (in the roof of building in faculty of science Ain Shams University, Cairo, Egypt) from ≈ 10 am to 4 pm every day over 5 d from 7 to 11 April 2019.

Weather data were recorded on an hourly basis from the Egyptian Meteorological Authority, Cairo, Egypt (about 1 km away from the experimental location) (Appendices A, Table A.3, supplementary data).

### 2.4 Data analysis

The interactions between grain temperatures and exposure time and the expected effect on insect mortality were investigated using the degree-minute model.

Degree-minutes (DM) were calculated according to [4, 5]:

DM = (T-B) × M

Where DM is the degree-minute value, *T* is the temperature (°C), *B* is the minimum temperature for killing *S. oryzae* (40 °C in this study), and *M* is the time (min).

## Ethics Statement

These data are a primary data and does not include any human subjects, animal experiment, or social media platforms.

## CRediT authorship contribution statement

**Shams Fawki:** Conceptualization, Methodology, Formal analysis, Visualization, Writing – original draft, Writing – review & editing. **Ayat Yousery:** Methodology, Visualization, Writing – review & editing.

## Declaration of Competing Interest

The authors declare that they have no known competing financial interests or personal relationships which have or could be perceived to have influenced the work reported in this article.

## Appendix Supplementary materials

Image, application 1
