# Supplementary material for: Dataset of thermal behaviour and weather data of thermal disinfestation of Sitophilus oryzae in plastic bags using solar heating
Source: Data Brief. 2022 Mar 7;42:108029. doi: 10.1016/j.dib.2022.108029 (PMC8958540; doi:10.1016/j.dib.2022.108029)
Supplement: Supplementary file 1 [file mmc1.pdf]

Journal of Stored Products Research 96 (2022) 101941

Contents lists available at ScienceDirect

Journal of Stored Products Research

journal homepage: www.elsevier.com/locate/jspr

Control of Sitophilus oryzae (Coleoptera: Curculionidae) in bags of wheat using solar radiation

S. Fawki [a,*], P.G. Fields [b], F. Jian [c], A. Yousery [a]

[a] Entomology Department, Faculty of Science, Ain Shams University, Abbasiya, 11566, Cairo, Egypt
[b] Morden Research and Development Centre, Agriculture and Agri-Food Canada, Winnipeg, MB, R3T 2M9, Canada
[c] Department of Biosystems Engineering, University of Manitoba, Winnipeg, MB, R3T 5V6, Canada

A R T I C L E I N F O

Keywords:
Sun exposure time
Rice weevil
Thermal control
Polyethylene bags
Greenhouse effect

A B S T R A C T

Solar heating disinfestation is a promising technique for low-income farmers in developing countries. Solar disinfestation of *Sitophilus oryzae* in wheat was investigated using clear and black polyethylene plastic bags and woven bags. Different amounts of wheat (16, 21, and 25 kg) with 12.2% moisture content (wet basis) were tested under two field conditions: 1) wheat bags were kept at the field with no stacking or mixing; 2) wheat bags were mixed at the end of the day and stacked during the night. The experiment was conducted in Canada and Egypt. Degree-minutes and time above 40 °C for each treatment were also calculated. Wheat inside clear polyethylene bags heated up to a temperature range of 40–55 °C, which is considered lethal to many stored-grain insects, including *S. oryzae*. Mortality of adult *S. oryzae* was 67.6 ± 30% for clear plastic bags and 4.3 ± 0.8% for woven plastic bags. In general, degree-minutes and time above 40 °C inside 16 kg of wheat bags were significantly higher than bags with 21 or 25 kg of wheat.

1. Introduction

Cereals, oilseeds and legumes are vital components of human food and animal feeds (Bendinelli et al., 2020). As the human population increases, food demand increases year by year. For developing countries, increasing grain production is critical to maintaining local market needs. Grain production significantly depends on high-quality postharvest storage. Unfortunately, many of these countries have inadequate postharvest facilities and technologies (Kumar and Kalita, 2017) to control insect infestation and high infestation potential due to high humidities and temperatures (Manandhar et al., 2018; Sallam, 2000). In some places in Africa, insect pests were responsible for up to 92% postharvest loss (Sallam, 2000; Yan et al., 2014).

Sitophilus oryzae (L.) (Coleoptera: Curculionidae) is one of the most destructive insect pests to wheat and other cereals (Rita Devi et al., 2017; Yan et al., 2014). *Sitophilus oryzae* is a primary pest of the whole kernels of grains. Adult feeding and larval activity inside kernels cause considerable grain weight loss, secondary pest infestation, and fungus growth (Athanassiou et al., 2017). The optimal conditions for *S. oryzae* development are 25–30 °C and ≈75% r.h. (Rita Devi et al., 2017; Singh et al., 1974). *Sitophilus oryzae* adults start mating after 3.6 days of emergence (Rita Devi et al., 2017), thus providing time for starting an appropriate control to prevent population growth. The total life cycle of *S. oryzae* is 39–114 days depending upon food type, humidity, and temperature (Okram and Hath, 2019; Rita Devi et al., 2017). *Sitophilus oryzae* adult longevity is relatively long compared to other stored grain insects. Male longevity is about 61 d, and female longevity is about 83 d. The fecundity rate is about 54 eggs/lifetime (Rita Devi et al., 2017).

Grain production in many developing countries mainly relies on small farmers and small-scale postharvest processing industries (Bendinelli et al., 2020; Manandhar et al., 2018). For example, the government in Egypt is the main purchaser for local wheat (about 37%), and the remaining wheat is consumed on-farm as human food or animal feed (McGill et al., 2015). This sector mainly uses aluminum phosphide as a stored-grain pest control fumigant. Fumigation is an inexpensive and convenient method, but it has many disadvantages compared to other control methods. There is growing insecticide resistance in insect populations to phosphine (Venkidusamy et al., 2017; Zettler and Keever, 1994). Fumigation can be dangerous if the label instructions are not followed, and can fail due to poor sealing (Rajendran and Sriranjini, 2007). Therefore, simple, effective, inexpensive, and nontoxic techniques are needed.

As an alternative, heat disinfestation of stored grain insects has been examined in many studies (Abdullahi et al., 2019; Beckett et al., 1998;

* Corresponding author.,
E-mail addresses: shfawki@sci.asu.edu.eg, shfawki@hotmail.com (S. Fawki).

https://doi.org/10.1016/j.jspr.2022.101941
Received 5 July 2021; Received in revised form 6 January 2022; Accepted 8 January 2022
Available online 22 January 2022
0022-474X/Crown Copyright © 2022 Published by Elsevier Ltd. All rights reserved.

Bingham et al., 2017; Chauhan and Ghaffar, 2002; Fawki et al., 2014; Lale and Vidal, 2003; Tang, 2007). A wide range of simple and advanced techniques have been used in different areas of the world. Heat control is very promising, especially against the stored-grain insects with immature stages develop internally inside the grains, such as *Rhyzopertha dominica* (F.) and *S. oryzae* (Beckett et al., 1998). Different studies showed that 100% mortality of *S. oryzae* adults was achieved within days to hours at 40–48 °C, hours to minutes at 48–55 °C and minutes to seconds at 55–66 °C (Beckett et al., 1998; Fields, 1992; Murdock and Shade, 1991; Yan et al., 2014). Generally, similar temperatures and durations as outlined above should be enough to kill most stored-product insects (Fields, 1992).

Solar heating has been used as a thermal control method against stored-product insects. Many studies have shown that solar radiation techniques are capable of raising the temperature of stored food items to lethal temperatures of many insect and arthropod pests (Abdullahi et al., 2019; Chauhan and Ghaffar, 2002; Fawki et al., 2014; Kitch et al., 1992; Lale and Vidal, 2003; Muhammad et al., 2006; Murdock and Shade, 1991; Ntoukam et al., 1997). Other studies also revealed that solar radiation and thermal control is very promising to control museum insects in an integrated pest management program (Brokerhof, 2003; Strang, 2012, 2014; Strang and Kigawa, 2009). Different solar heating systems have been developed to control insect pests; including metal boxes, plastic sheets, plastic bags, solar pillows, storage bins and many others (Abdullahi et al., 2019; Fawki et al., 2014; Kitch et al., 1992; Muhammad et al., 2006; Strang, 2012).

Some of the heat treatment methods are not appropriate for small-scale farmers, such as structure heat disinfestation and big silos heating control (Beckett et al., 2007; Muhammad et al., 2006). However, some of the solar methods show promise (Kitch et al., 1992; Murdock and Shade, 1991; Ntoukam et al., 1997; Strang, 2014). The adoption of such techniques by small-scale farmers and grain industries will minimize the hazards of using fumigants and other chemical control methods. Most of these techniques used black plastic sheets covered with clear plastic sheets. These studies depend mainly on a small grain capacity (1–2 kg) or small museum subjects. Even when a larger grain capacity (50 kg) was used, the grains were subjected to solar heating in a thin layer of grains without considering grain thickness as a parameter. Grains are considered as a good insulator as it has low thermal conductivity causing grain bulks to change temperature slowly (Jian et al., 2013; Muhammad et al., 2006).

Therefore, our objective was to test the effectiveness of plastic polyethylene bags as solar heat collectors to control stored product insects in different amounts of grain bulks. Solar disinfestation using polyethylene bags on the fields is affected by many factors. For the current study, grain thickness and mass, bag materials, and stacking vs non-stacking of the bags were investigated. We tested three different bag materials; clear polyethylene, black polyethylene, and woven plastic polyethylene, and different quantitates of grain (16, 21, and 25 kg). Finally, we tested the effect of mixing grain in bags and stacking bags at the end of the day, as grain retained heat when the bags were stacked together.

2. Materials and methods

2.1. Bags and temperature measurements

The wheat used in the Canadian tests was Canadian hard red spring wheat (SY Slate variety, Pitura Seeds) with 12.2 ± 0.0% m.c.. Wheat was placed into either clear bags (Ploy Bags, Uline, Canada, 76.2 μm thickness) or black bags (Ploy Bags, Uline, Canada, 152.4 μm thickness). For this test, bags had 16, 21 or 25 kg of wheat. Grain bags were placed in open-top square plywood boxes made from 12.5 mm thick plywood with a dimension of 45 cm length and width and different heights: 9, 13, and 15 cm. Open-top boxes were used to collect solar radiation and maintain a uniform shape and heights of the grain bulk (different heights, 9, 13, and 15 cm, for 16, 21, and 25 kg of grains, respectively). Another advantage of using a wooden substrate is to eliminate the variation of the ground.

The grain temperature was measured using data loggers (HOBO Thermocouple Data Loggers, Onset Computer Corporation, Bourne, MA, USA) every 15 min. In each bag, six thermocouples were inserted through the centre of the top surface of the grain bag into the grain mass and equally distributed in the grain mass in the vertical direction. The distances between thermocouples were calculated according to the grain height in bags, and the locations of the six thermocouples were fixed by heat-shrink tubing. For example, thermocouples were 0, 0, 2.25, 4.5, 6.75 and 9 cm depth in the 16 kg wheat bag in the vertical direction at the following locations: the outer surface of the bag, just beneath the bag's surface from the inner side, between top and middle, the middle, between middle and bottom, and the bottom; respectively. For the 21 kg bags, thermocouples were 0, 0, 3.25, 6.5, 9.75 and 13 cm depth in the vertical direction at the following locations: the outer surface of the bag, just beneath the bag's surface from the inner side, between top and middle, the middle, between middle and bottom, and the bottom; respectively. For the 25 kg bags, thermocouples were 0, 0, 3.75, 7.5, 11.25 and 15 cm depth in the vertical direction at the following locations: outer surface of the bag, just beneath the bag's surface from the inner side, between top and middle, the middle, between middle and bottom, and the bottom; respectively. The thermocouple at the grain surface was taped to the surface of a square plastic disc, so the heat-shrink tubing was fixed. The temperature cables were removed at the end of the day and reinserted at the beginning of each day.

2.2. Preliminary study in Canada: clear and black bags

Trials were conducted over 7 d from 28 June to July 12, 2018 in Winnipeg, Canada, for 2.5 h between 11:00–19:00 (times differed from day to day). There were 16, 21, and 25 kg of wheat in each bag. The main objective for this treatment was to find whether clear or black bags collect more solar radiation and maintain grain temperature. Therefore, there was only one replicate of each clear and black bag at each grain mass. Grain temperatures were measured using data loggers as mentioned in 2.1 section. The ambient temperature was recorded using a Campbell Scientific weather station (Campbell Scientific, Logan, Utah, USA), which was set close to the bags in the field.

2.3. Field study in Canada

2.3.1. Grain bulk with stacking (Canadian test 1)

This test was designed to determine if unstacked bags during the day, then stacked as a group in an insulated container during the night, would maintain higher grain temperatures more than single unstacked bags. As clear polyethylene plastic bags had a higher temperature than black bags (this was shown in the preliminary study), only clear bags were used for this test. The grain (≈255 kg) was extensively mixed in a rotating drum to give a uniform moisture content (m.c.) of the wheat between bags. The m.c. of the wheat after loading into the bags were 12.5 ± 0.0%. The grain moisture content was measured by using ASABE standard at 130 °C for 19 h (ASABE, 2016).

On the day of the test, plywood sheets were laid down on a flat grass lawn with no shading to cover the whole experimental area (Fig. 1). The bags holding three different grain masses were evenly distributed over the plywood sheets, 45 cm apart from each other (Fig. 1). The bags holding 16, 21, or 25 kg were placed in wooden boxes (45 × 45 cm) (the grain had the same height as the inner height of the boxes), and another treatment with 21 kg bag was not placed in a wooden box. There were three replicates for each treatment. The solar exposure was conducted from ≈11 a.m. to 8 p.m. for 5 d in July 2018. At the end of each day, temperature cables were removed, bags were turned to mix the grain without opening the bags, and then the bags were stacked in foam boxes during the night to retain the heat. There were four foam boxes, three

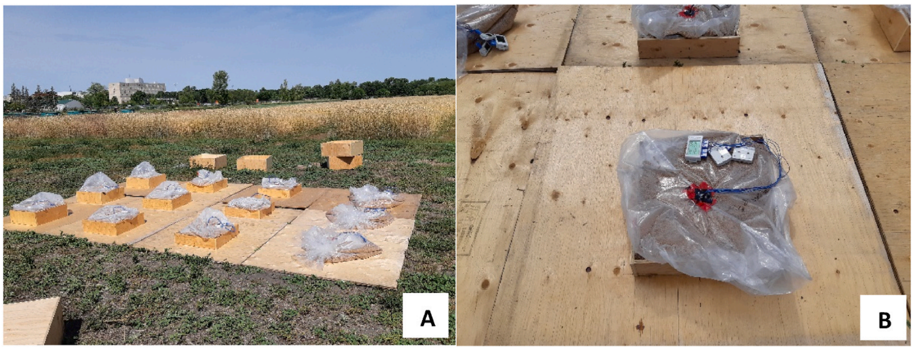

Fig. 1. Using solar radiation to heat wheat in bags, Canadian trials: a) field set up during day; and b) enlarged view of treatments.

large and a small one. The large box external dimensions were 87.6 × 110.5 × 81.3 cm, and the small foam box external dimensions were 71.1 × 71.1 × 71.1 cm. The foam boxes were made of extruded polystyrene foam Insulation (Styrospan^{Tm}, Dow Chemical, Mississauga, Ontario, Canada), and the thickness of the walls of the foam boxes was ≈14 cm. The thermal resistance (R) value of the foam is 5.3 $m^{2\circ}C/W$. The large foam boxes were used to store the bags holding 21, 25 kg wheat in a wooden box and 21 kg of wheat not in a wooden box. The small foam box was used for the 16 kg wheat in a wooden box.

Campbell Scientific weather station was set at the experiment site. Weather data were collected every 15 min. The collected data were temperature, humidity, air pressure, solar radiation, precipitation, wind speed, and wind direction (Appendices A; Table A.1) (Fawki and Yousery, 2021).

Grain moisture content and germination were measured before and after the treatment by taking three samples from each bag before the treatment. Germination was measured by following the method reported by Jian et al. (2009). To measure the germination, 25 seeds were placed onto a wet filter paper located in a 90 mm Petri dish. Germination was determined after 7 d incubation at room temperature (25 ± 2 °C).

2.3.2. Grain bulk without stacking (Canadian test 2)

The protocol of this test was the same as that mentioned above, except that the bags were left on the field continuously over 6 d in August 2018. No stacking or mixing was done, and thermocouples were not moved overnight. At the end of each day, all bags were covered with a wooden plywood box to prevent any disturbance from wild animals. The weather collected data were temperature, humidity, air pressure, solar radiation, precipitation, wind speed, and wind direction (Appendices A; Table A.2) (Fawki and Yousery, 2021).

2.4. Field study in Egypt

2.4.1. Grain bulk with stacking

Given that the data from Canada showed the highest temperatures were obtained from the smallest grain bag (16 kg), only 16 kg wheat held in clear bags and woven plastic bags were tested in Egypt. Before the test, wheat was extensively mixed using a shovel.

Three treatments were conducted: clear bags (Makhlouf Group, Cairo, Egypt, 50.8 μm thickness), woven plastic bags (Makhlouf Group, Cairo, Egypt), and control in clear bags that remained indoors. Each treatment had three replicates. The experiment was conducted on the roof of a building in Cairo, Egypt, in April 2019. The bags were exposed to the sun from ≈10 a.m. to 4 p.m. each day over 5 d. The methods of stacking, mixing, packing in foam boxes, and temperature measurement were the same as those conducted in Canada (Section 2.3.1.) except that a plywood sheet (57 cm in both length and width) was located under each wood box holding grain bags. The outer dimensions of the foam boxes used were 55 × 55 × 72 cm. The wall of the box was made from Styrofoam with 13 cm thickness. The cover of the foam box has 5 cm thickness but was made from the same materials as the walls. The wheat in bags (including the bags for the control) was mixed (turned 4–5 times) at the end of every day and kept separately in a lab (30 ± 2 °C and 35 ± 5% r.h.). Hourly weather data collected by the Egyptian Meteorological Authority, Cairo, Egypt (about 1 km away from the experimental location) were used (Appendices A; Table A.3) (Fawki and Yousery, 2021).

2.4.2. *Sitophilus oryzae* adult mortality

The Egyptian trial also measured the survival of *S. oryzae* adults in vials placed in the bags. To get the adults of a similar age, 500 adults were added to 3 kg of whole wheat, purchased from a local market, held in a plastic jar covered with a double layer of muslin cloth which was tied by rubber bands. The insects and feed were kept in the dark in an incubator at 28 ± 2 °C and 35 ± 2% r.h. One hundred adults of *S. oryzae* (0–12 d old) were generally selected and placed in a 15 ml Falcon vial (1.5 cm in diameter and 15 cm length) with 15 g wheat. One vial was placed at the centre of each bag and retrieved at the end of the treatment, and the number of live and dead adults were counted. Adult insects were considered dead when there was no sign of movement upon being touched with a fine brush.

2.5. Data analysis

One-way ANOVA was conducted using the Tukey-Kramer HSD test to compare the degree-minutes and the time above 40 °C among different treatments. When the assumption of homogeneity of the variance was not fulfilled even after transformation, a Welch ANOVA or Wilcoxon/Kruskal-Wallis tests (rank sums) was applied. Germination and m.c. among different treatments were compared by using one-way ANOVA or Wilcoxon/Kruskal-Wallis tests. The data of m.c. was significant in Canada grain bulk size and non-stacking experiment; thus, a Dunnett's test was applied. The degree-minute model was used to investigate the relationship among temperature, exposure time, and insect mortality. Degree-minutes (DM) were calculated according to (Subramanyam et al., 2002; Tang, 2007):

$$DM = (T - B) \times M$$

Where DM is degree-minute value, T is the temperature (°C), B is the minimum temperature for killing *S. oryzae* (40 °C in this study), and M is the time (min).

3. Results

3.1. Preliminary test: Canada: clear vs black bags

The grains in clear bags were warmer than grains in black plastic bags. Grain temperatures inside the clear bags at the top, middle and bottom of the bags were 3–4 °C higher than that of grain at the corresponding locations in the black bags (Table 1).

3.2. Field study in Canada

3.2.1. Grain bulk with stacking (Canadian test 1)

The 16 kg bulk of wheat heated up to more than 40 °C (temperatures that can be lethal to *S. oryzae*) at some locations in the clear plastic bags (Fig. 2,Figs. A1, A2, A3 & A4, in the supplementary materials) (Fawki and Yousery, 2021). The grain at the top layer had higher temperatures than that at the lower part (middle and bottom) for all three of the grain bulks layers (Fig. 2, Figs. A1, A2, A3 & A4, in the supplementary materials) (Fawki and Yousery, 2021). Temperatures in the top layers reached up to 55 °C (Fig. 2). Even though the wheat at the bottom of the bag had a lower temperature, it reached more than 40 °C after 3 d in the 16 kg bulk (Fig. 2, Fig. A1 in the supplementary materials) (Fawki and Yousery, 2021). The maximum ambient air temperature was 32 °C, and the highest solar radiation was 0.83 (Mj/m^2) during the experimental period (Fig. 3).

There was a significant difference in degree-minutes (DM) (Table 2, One-way ANOVA: $F_{3,8} = 4.5996$, $P = 0.0375$) and time above 40 °C at the bottom (Table 2, One-way ANOVA: $F_{3,8} = 14.4864$, $P = 0.0013$) between different wheat bulks in all treatments. The DM in the 16 kg bulk were significantly higher than that in the 21 kg bulk (without wooden box) (Tukey-Kramer HSD test: $P = 0.0381$) but were not significantly different from either the DM of 21 or 25 kg treatments (Table 2) (Tukey-Kramer HSD test: $P = 0.4618$ and $P = 0.0807$, respectively). The time at 40 °C or higher at the bottom of the 16 kg wheat was significantly longer than that in 21 and 25 kg bulk (Table 2). Solar heating had no significant effect on grain germination (Table 2) and grain m.c. (Table 2).

3.2.2. Grain bulk size without stacking (Canadian test 2)

The bottom temperatures reached 40–43 °C in the 5th and 6th days in 16 kg treatment (Fig. 4, Fig. A5, in the supplementary materials) (Fawki and Yousery, 2021). Almost none of the other treatments bottom reached that high of a temperature, except one replicate in the 21 kg treatment (Fig. 4, Figs. A5, A6, A7 & A8 in the supplementary materials) (Fawki and Yousery, 2021). Grains in top layers gained heat rapidly during the daytime and lost their heat rapidly during the night, compared to grains at the bottom (Fig. 4, Figs. A5, A6, A7 & A8 in the supplementary materials) (Fawki and Yousery, 2021). The grain temperature fluctuation pattern follows the air temperature patterns (Figs. 4 and 5, Figs. A5, A6, A7 & A8 in the supplementary materials) (Fawki and Yousery, 2021).

There was a significant difference in DM between different

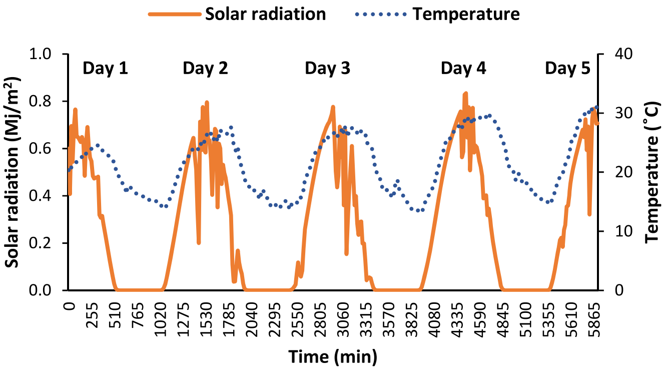

Fig. 3. Solar radiation (Mj/m^2) and ambient temperatures (°C) during the field trial in Canada from 26 to July 30, 2018 (5 d) **(Canadian test 1).**

treatments (Table 3, One-way ANOVA: $F_{3,7} = 8.5932$, $P = 0.0096$). DM of 16 kg grain bulks is significantly different from that of 21, 25 and 21 kg (not in the wooden box) (Tukey-Kramer HSD *t*-test: $P = 0.0259$, $P = 0.0079$, $P = 0.0207$, respectively).

Solar heating had no significant effect on grain germination (Table 3). On the other hand, there was a significant effect on grain m.c. % (Table 3). After the solar radiation, the m.c. of 16, 21, and 25 kg of grain bulk was significantly lower than that of the initial m.c. of wheat (Dunnett's test: $P = 0.0001$, $P = 0.0001$ and $P < 0.0001$, respectively). The treatment of 21 kg of wheat (not in the wooden box) had m.c. higher than that of the initial m.c. of wheat (Dunnett's test: $P < 0.0001$).

3.3. Field study in Egypt: bag type with stacking, and Sitophilus oryzae mortality

There was no significant difference in DM between clear and woven bag treatments (Table 4, Matched pairs: $t = -2.54341$, $df = 2$ $P = 0.1260$). On the other hand, time above 40 °C for clear bags was significantly higher than that of woven bags (Table 4, Matched pairs: $t = -4.51773$, $df = 2$, $P = 0.0457$). The temperature profile of clear bags was significantly higher than that of the woven one (Figs. 6 and 7, Fig. A9 & A10, in the supplementary materials) (Fawki and Yousery, 2021). In clear bags, the bottom reached 40 °C or above starting from the second day onwards (Fig. 6, Fig. A9, in the supplementary materials) (Fawki and Yousery, 2021), while in the woven bags, the bottom reached temperatures around 40 °C on the fifth day (Fig. 7, Fig. A10, in

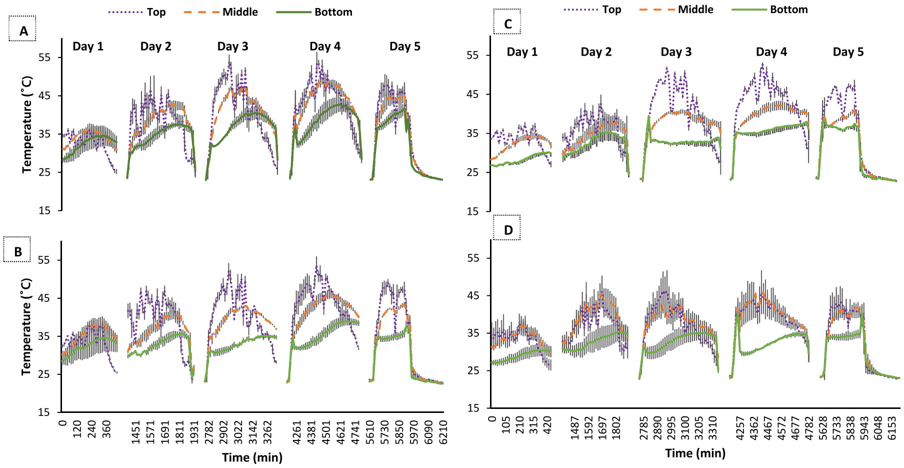

Fig. 2. Temperatures (mean ± *SE*) of the wheat bulk in clear bags in Canada from 26 to July 30, 2018 **(Canadian test 1)**. A, B, C and D for 16 and 21, 25 kg of wheat and 21 kg of wheat without wooden box, respectively. The grain was mixed and stacked in individual insulated foam boxes during night and exposed to sun during day. Temperature was not measured during night.

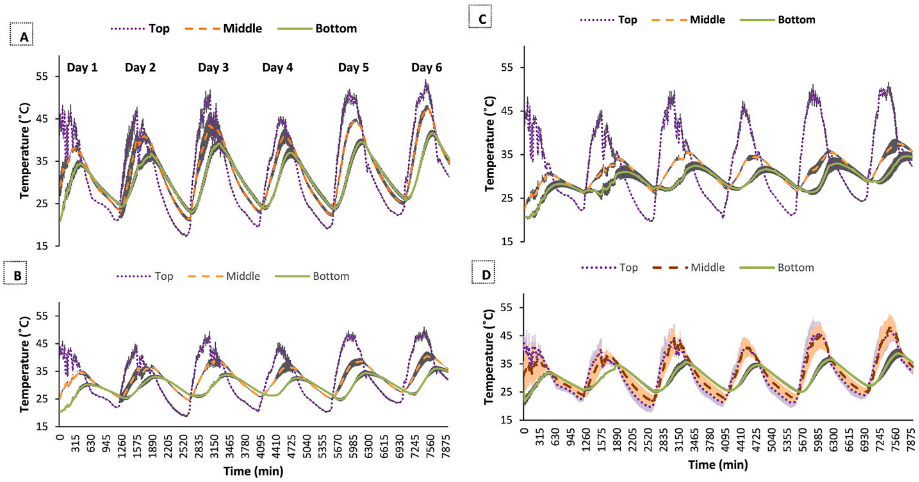

Fig. 4. Temperatures (mean ± *SE*) in wheat bulk in clear bags in Canada from 7 to August 12, 2018 **(Canadian test 2)**. A, B, C and D for 16 and 21, 25 kg of wheat and 21 kg of wheat without wooden box, respectively. The grain was not mixed and held in separate wooden boxes during the day and night.

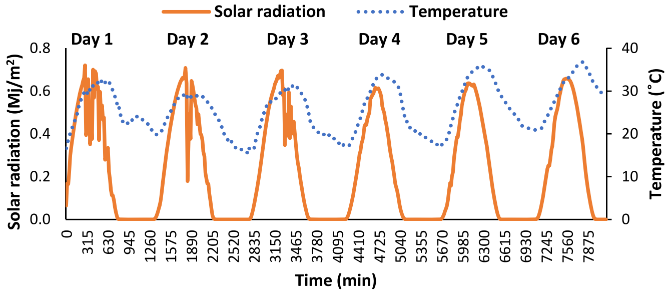

Fig. 5. Solar radiation (Mj/m^2) and ambient temperatures (°C) during the field trial in Canada from 7 to August 12, 2018 (6 d) **(Canadian test 2)**.

Table 1
Temperatures at the top, middle and bottom of clear and black plastic bags holding 16, 21, or 25 kg wheat in the preliminary test in Canada. Data show the temperatures after 2.5 h of sun exposure every day over 7 d of the experiment.

Position in bag	Clear Bags (°C, Mean ± *SE*)	Black Bags (°C, Mean ± *SE*)	Difference[a] (°C, Mean ± *SE*)	*P*-value[b]
Top	39.6 ± 2.2	35.9 ± 2.2	3.7 ± 1.0	0.0165
Middle	36.4 ± 1.8	33.3 ± 2.4	3.1 ± 0.9	0.0268
Bottom	33.3 ± 1.8	29.3 ± 2.4	4.0 ± 0.8	0.0134

[a] Temperature difference between in clear and black plastic bags at the corresponding locations.
[b] Paired *t*-test, n = 7.

the supplementary materials) (Fawki and Yousery, 2021). Both clear bags and woven bags had temperatures higher than the air temperature, but the clear ones were more efficient in heating. The temperature increased by 15–20 °C compared to air temperature (Figs. 6–8).

Solar heat treatment had no significant effect on grain germination (Table 4). However, there was a significant effect on grain m.c. (Table 4). After heat treatment, m.c. of grains in clear bags were significantly higher than the control (Dunnett's test: $P = 0.0065$), while the m.c. of grains in woven bags did not differ from the control treatment (Dunnett's test: $P = 0.1192$).

Adult mortality of *S. oryzae* was significantly different between treatments (Table 4, Wilcoxon/Kruskal-Wallis tests: $X^2 = 6.7126$, $df = 2$, $P = 0.0349$). The overall adult mortality of *S. oryzae* was 68 ± 30% inside clear bags, 4.3 ± 0.8% in woven bags and 0 ± 0% in the control treatment with no solar heating. In clear bag treatment, there was significant variation in adult mortality between the three replicates: 94.9, 100 and 7.9% in replicates one, two and three, respectively. At the same time (on day five), the range of temperature at the bottom in the clear bags was 40.0–43.6, 40.0–44.6 and 36.5–38.8 °C for replicates one, two, and three, respectively (Fig. 6, Fig. A9, in the supplementary materials) (Fawki and Yousery, 2021). There was the lowest mortality in replicate 3, which also had the lowest temperature.

Table 2
Time/day above 40 °C (min) at the bottom, degree-minutes/day, germination and m.c. of 16, 21 and 25 kg wheat of plastic bags inside wood boxes and another 21 kg of wheat in a plastic bag without a wood box exposed to solar radiation over 5 d in Canada (Canadian test 1).

Wheat mass (kg)	Treatment	Time/day above 40 °C (min, mean ± *SE*)[a]	Degree-minutes/day (°C-min, mean ± *SE*)[b]	Germination (%, mean ± *SE*)[c]	m.c. (%, mean ± *SE*)[d]
16	before exposure[e]	–	–	97.3 ± 1.3 **a**	12.48 ± 0.0 **a**
16	Wooden box	2.5 ± 0.6 **a**	1043 ± 133 **a**	97.3 ± 0.8 **a**	12.5 ± 0.0 **a**
21	Wooden box	0.6 ± 0.2 **b**	781 ± 139 **ab**	97.3 ± 0.9 **a**	12.5 ± 0.0 **a**
25	Wooden box	0.1 ± 0.1 **b**	554 ± 69 **ab**	98.7 ± 0.8 **a**	12.5 ± 0.01 **a**
21	Not in wooden box	0.1 ± 0.01 **b**	463 ± 129 **b**	96.0 ± 1.3 **a**	12.5 ± 0.01 **a**

Different letters indicate significant differences between treatments ($P < 0.05$).
[a] For a given row, means followed by different letters are significantly different, One-way ANOVA: $F_{3,8} = 14.4864$, $P = 0.0013$.
[b] For a given row, means followed by different letters are significantly different, One-way ANOVA: $F_{3,8} = 4.5996$, $P = 0.0375$.
[c] For a given row, means followed by different letters are significantly different, One-way ANOVA: $F_{4,10} = 0.9691$, $P = 0.4658$.
[d] For a given row, means followed by different letters are significantly different, One-way ANOVA: $F_{4,10} = 0.7558$, $P = 0.5768$.
[e] Germination and m.c. of wheat samples before the sun exposure.

4. Discussion

The clear bags were more efficient than black ones in heating grains. This heating is probably due to the greenhouse effect (Baneshi et al., 2020; Bastien, 2015; MWPS-22, 1980; Stapleton, 1996; Strang and Kigawa, 2009). As the clear plastic allows the penetration of shortwave solar radiation inside the bags and block longwave radiation from the

Table 3
Time/day above 40 °C (min) at the bottom, degree-minutes/day, germination and m.c. of 16, 21 and 25 kg wheat in plastic bags inside wood boxes and another 21 kg of wheat in a plastic bag without a wood box exposed to solar radiation over 6 d in Canada (Canadian test 2, without stacking). All treatments were kept continuously in the filed during the experiment.

Wheat mass (kg)	Treatment	Time above 40 °C/day (min, mean ± *SE*)[a]	Degree-minutes/ day (°C-min, mean ± *SE*)[b]	Germination (%, mean ± *SE*)[c]	m.c. (%, mean ± *SE*)[d]
16	before exposure[e]	–	–	100.0 ± 0.0 **a**	12.8 ± 0.0 **a**
16	Wooden box	0.5 ± 0.3	1359 ± 218 **a**	97.8 ± 0.9 **a**	12.5 ± 0.0 **b**
21	Wooden box	0	714 ± 114 **b**	98.7 ± 0.8 **a**	12.5 ± 0.0 **b**
25	Wooden box	0	548 ± 60 **b**	96.9 ± 1.2 **a**	12.5 ± 0.02**b**
21	Not in wooden box	0.1 ± 0.1	694 ± 57 **b**	94.7 ± 2.1 **a**	13.1 ± 0.01**b**

Different letters indicate significant differences between treatments ($P < 0.05$).
[a] For a given row, means followed by different letters are significantly different.
[b] For a given row, means followed by different letters are significantly different, One-way ANOVA: $F_{3,10} = 8.5932$, $P = 0.0096$.
[c] For a given row, Wilcoxon/Kruskal-Wallis tests: $X^2 = 8.4423$, $df = 4$, $P = 0.0767$.
[d] For a given row, means followed by different letters are significantly different, One-way ANOVA: $F_{4,10} = 104.4942$, $P < 0.0001$.
[e] Germination and m.c. of wheat samples before the sun exposure.

Table 4
Time/day above 40 °C (min) at the bottom, degree-minutes/day, germination and m.c. of 16 kg of wheat in plastic and woven plastic bags inside wood boxes after exposure to sun radiation over five days, Stacking and mixing experiment (Egypt).

Treatment	Time/day above 40 °C (min, mean ± *SE*)[a]	Degree-minutes/day (°C-min, mean ± *SE*)[b]	Germination (%, mean ± *SE*)[c]	m.c. (%, mean± *SE*)[d]
Indoors	–	–	70.7 ± 8.7 **a**	10.8 ± 0.0 **a**
Clear	5.3 ± 1.3 **a**	909 ± 197 **a**	67.6 ± 2.5 **a**	11.5 ± 0.1 **b**
Woven	0.6 ± 0.3 **b**	235 ± 69 **a**	72.9 ± 3.8 **a**	11.1 ± 0.2 **a**

Different letters indicate significant differences between treatments ($P < 0.05$).
[a] For a given row, means followed by different letters are significantly different, Matched pairs: $t = -4.51773$, $df = 2$, $P = 0.0457$.
[b] For a given row, means followed by different letters are significantly different, Matched pairs: $t = -2.54341$, $df = 2$ $P = 0.1260$.
[c] For a given row, means followed by different letters are significantly different, One-way ANOVA: $F_{2,6} = 0.2193$, $P = 0.8092$.
[d] For a given row, means followed by different letters are significantly different, Welch ANOVA: $F^2 = 60.2286$, $P = 0.0060$.

grains (absorber material), the retained solar energy increases grain temperature (Baneshi et al., 2020; Lamont, 1996; MWPS-22, 1980; Stapleton, 1996; Taki et al., 2018). Generally, using clear plastic films have been successfully used in solar disinfestation for both soil and museum pests (Chase et al., 1999; Strang and Kigawa, 2009). Similar findings were recorded for soil disinfestation since the clear polyethylene film was more efficient in soil heating than the black ones.

On the other hand, innovative and simple solar heating techniques were developed in Canada for thermal disinfestation for museum objects (Strang, 2012, 2014; Strang and Kigawa, 2009). Among these techniques, a solar plastic capsule and a solar disinfestation plastic frame were used. Each technique depends on raising temperatures (up to 65 °C) by clear polyethylene plastic containers to kill insect pests, especially in ambient cold environmental conditions (Strang, 2012, 2014). Another study also revealed that solar heaters made of a combination of black and clear plastic sheets were effective to eliminate any insects from stored grains (Chinwada and Giga, 1996). Our results also show that clear polyethylene bags are the efficient heating system for grains to reach lethal insect temperatures up to 55 °C.

Our work revealed that the lowest grain bulk tested, 16 kg, had the highest temperature profile compared to other treatments, especially the bottom of the bags. The smaller grain bulks, the higher the possibility of all grains will be heated, and the amount of cool grain will be reduced, where the insects can take as a shelter. During the daytime, the temperatures of the top layer from top to middle-bottom positions had higher temperatures than that of the grain bottom, while at night, this trend is revised, and the bottom had higher temperatures than the top layer. Another solar disinfestation study using a steel bin reported a similar trend between the top and bottom layers during day and night (Muhammad et al., 2006). This thermal gradient is mainly due to the low thermal conductivity of wheat (Muhammad et al., 2006; MWPS-22, 1980) and heat loss that occurs across the clear plastic side during the night (Joudi and Farhan, 2014).

In our study, there was a trend that stacking the grain bags overnight increased the time at lethal temperatures at different locations inside the bags and the bottom of the bags compared to non-stacking treatments. This trend is observed in both Canada and Egypt data as the bottom temperature in the stacking experiment was 40 °C and above compared to lower temperatures in non-stacking treatment. Grains inside the bags are opaque materials that absorb solar radiation and have a high thermal capacity to retain heat (Arinze, 1978; MWPS-22, 1980). Under stacking condition, the grains act as a good insulator and minimizing conduction heat loss during nights (Al-Amri and Abbouda, 2013; Arinze, 1978). These results need to be confirmed by more data analysis and the development of a thermal model. On the other hand, data of DM supports those bags of small grain amount (16 kg) had higher DM than that of other treatments. However, it is not important as time above 40 °C, especially at the bottom, which is essential to cause almost 100% insect mortality. DM for non-stacking treatment was slightly higher than that of stacking one. An explanation for this could be that the last 3 d in the non-stacking experiment were sunny and had higher air temperatures compared to the stacking experiment. An indication for this the smooth peaks of total solar radiation compared to irregular peaks of the first three days.

All data from Egypt and Canada support the fact that the best exposure time is 2 h range before and afternoon. There was a synchronization between the highest temperature inside the bags and the highest total solar radiation in the middle of the day when the solar radiation is perpendicular to the earth surface. Another study also revealed that the temperature inside a greenhouse also increases by time from 9 a.m. to 12 p.m. then starts to decrease as the intensity of solar radiation decreases (Joudi and Farhan, 2014).

In our limited tests in Egypt with insects, the clear bags gave 69% mortality, whereas there was almost no mortality in the woven bags that producers and farmers usually use. Limited insect mortality data did not support the 100% insect control due to the low mortality in the third replicate, but the other thermal data gave promising results. The mortality experiment was conducted in the spring in Egypt when the temperature ranges from 26 to 32 °C. The effect of the heating capacity of the clear bags will be higher when used in typical summer weather conditions when the air temperatures reach up to 45–50 °C.

Solar heating treatment had no adverse effect on wheat grains quality, neither on seed germination nor grain m.c.. A slight increase in grain m.c. was observed in Egypt, which might result from increasing grain respiration due to the heating effect (Smith, 1969). The higher temperatures that are possible in the summer may affect germination

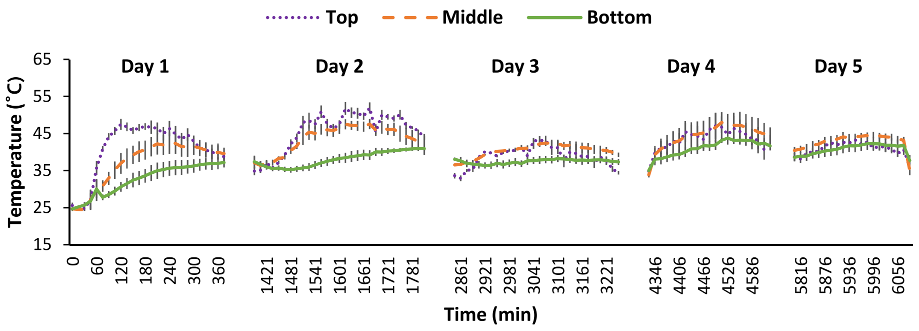

Fig. 6. Temperatures (mean ± *SE*) of 16 kg wheat bulk in clear bags in Egypt from 7 to July 11, 2019. The grain was mixed and stacked in insulated boxes for the night and in separate wooden boxes during the day.

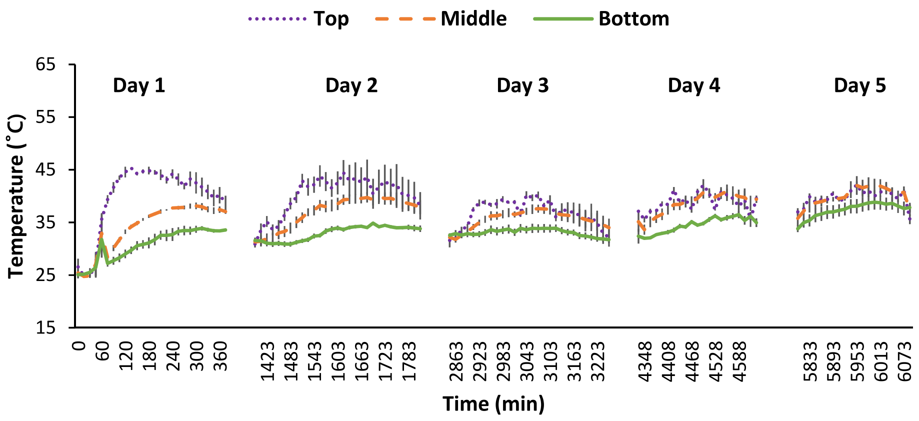

Fig. 7. Temperatures (mean ± *SE*) of 16 kg wheat bulk in woven bags in Egypt from 7 to July 11, 2019. The grain was mixed and stacked in insulated boxes for the night and in separate wooden boxes during the day.

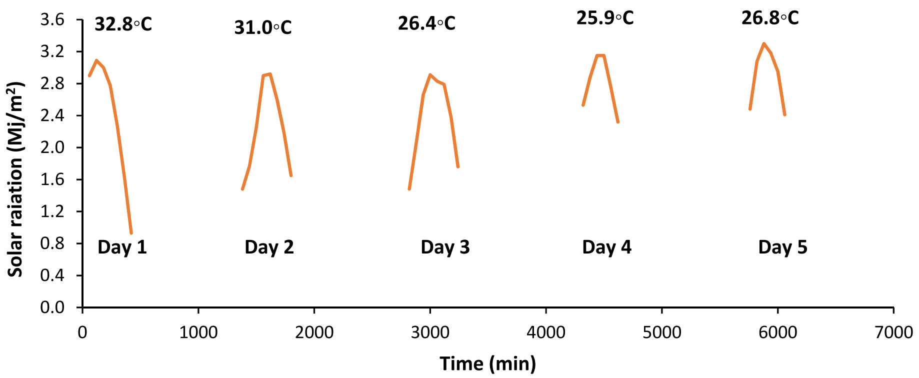

Fig. 8. Solar radiation (Mj/m^2), the maximum ambient temperature (°C) during the field trial in Egypt from 7 to July 11, 2019 (5 d).

and end-use quality, and further testing is needed.

In conclusion, grain temperatures increased by time and days and the clear polyethylene bags was able to raise grain temperatures by 15–20 °C above the ambient air temperatures. Heating the grains creates a heat gradient decreasing from the top layers (50–55 °C) to the bottom layers (40–45 °C). These temperature ranges are lethal for *S. oryzae* and many other stored-grain insects. The efficiency of solar heating system could be improved by two factors. The first is using a thermal blanket at night instead or beside stacking of the bags (Bastien, 2015; Taki et al., 2018). This insulation blanket will reduce the grain heat loss by convection and conduction (Kıyan et al., 2013). The second is to limit the opening and closing times of the thermal blanket to 2 h range before and after noon time. A 2-h-period before and after the noontime was previously recommended for seeds solar heating (Murdock and Shade, 1991; Ntoukam et al., 1997). Since the maximum solar transmission through a transparent material occurs when the solar radiation is perpendicular to the earth surface (Al-Amri and Abbouda, 2013; Bastien, 2015). This study also demonstrated the thermal migration through the grains in the vertical direction, while previous studies examined the solar heating on a horizontal direction (Chinwada and Giga, 1996; Kitch et al., 1992; Murdock and Shade, 1991; Ntoukam et al., 1997). The solar heating bags gave a promising result, although many crucial results are still unclear due to the lack of thermal models. Such models are essential for identifying the key parameters affecting the heating capacity of clear polyethylene bags.

CRediT author statement

Shams Fawki: Conceptualization, Methodology, Formal analysis, Visualization, Writing-original draft, Writing-review& editing. **Paul**

Fields: Conceptualization, Methodology, Formal analysis, Resources, Writing-review& editing. **Fuji Jian**: Conceptualization, Methodology, Writing-review&editing. **Ayat Yousery:** Methodology, Visualization, Writing-review&editing.

Declaration of competing interest

The authors declare that they have no known competing financial interests or personal relationships that could have appeared to influence the work reported in this paper.

Acknowledgements

We would like to thank the following people for technical assistance: Liam Carlin, Colin Demianyk, Kim Hamilton, Abubakr El-Sobky, and Ehab Sami. We also thank Walid Aboelsoud, Assistant professor, Mechanical Power Engineering, Faculty of Engineering, Ain Shams University, for assistance in data analysis and graphing. This work is supported by the Science and Technology Development Fund (STDF) [Short Term Fellowship (STDF-STF), grant number 25482, 2015], Cairo, Egypt.

Appendix A. Supplementary data

Supplementary data to this article can be found online at https://doi.org/10.1016/j.jspr.2022.101941.

References

Abdullahi, G., Muhamad, R., Dzolkhifli, O., Sinniah, U.R., 2019. Efficiency of cardboard solar heater boxes for disinfestations of stored grains against arthropod pest. Agric. Sci. Technol. 11, 247–256. https://doi.org/10.15547/ast.2019.03.043.

Al-Amri, M.S., Abbouda, K., 2013. Thermal control of stored grains insects by utilizing solar energy. Ama, Agric. Mech. Asia, Afr. Lat. Am. 44, 39–45.

Arinze, E.A., 1978. Solar Energy Absorption Properties of Some Agricultural Products, Agricultural and Bioresource Engineering. University of Saskatchewan, Saskatoon, Saskatchewan, Canada.

ASABE, 2016. Moisture Measurement –Unground Grain and Seeds, ASABE Standard. American Society of Agricultural and Biological Engineers, St. Joseph, MI.

Athanassiou, C.G., Kavallieratos, N.G., Campbell, J.F., 2017. Competition of three species of *Sitophilus* on rice and maize. PLoS One 12, e0173377. https://doi.org/10.1371/journal.pone.0173377.

Baneshi, M., Gonome, H., Maruyama, S., 2020. Wide-range spectral measurement of radiative properties of commercial greenhouse covering plastics and their impacts into the energy management in a greenhouse. Energy 210, 118535. https://doi.org/10.1016/j.energy.2020.118535.

Bastien, D., 2015. Methodology for Enhancing Solar Energy Utilization in Solaria and Greenhouses. Concordia University.

Beckett, S., Fields, P., Subramanyam, B., 2007. Disinfestation of stored products and associated structures using heat. In: Tang, J., Mitchmam, E., Wang, S., Lurie, S. (Eds.), Heat Treatments for Postharvest Pest Control: Theory and Practice. CAB International, Oxon, United Kingdom, pp. 182–236.

Beckett, S.J., Morton, R., Darby, J.A., 1998. The mortality of *Rhyzopertha Dominica* (F.) (Coleoptera : Bostrychidae) and *Sitophilus oryzae* (L.) (Coleoptera : Curculionidae) at moderate temperatures. J. Stored Prod. Res. 34, 363–376.

Bendinelli, W.E., Su, C.T., Péra, T.G., Caixeta Filho, J.V., 2020. What are the main factors that determine post-harvest losses of grains? Sustain. Prod. Consum. 21, 228–238. https://doi.org/10.1016/j.spc.2019.09.002.

Bingham, A.C., Subramanyam, B., Mahroof, R., Alavi, S., 2017. Development and validation of a model for predicting survival of young larvae of *Tribolium castaneum* exposed to elevated temperatures during heat treatment of grain-processing facilities. J. Stored Prod. Res. 72, 143–152. https://doi.org/10.1016/j.jspr.2017.04.008.

Brokerhof, A.W., 2003. The solar tent—cheap and effective pest control in museums. AICCM Bull. 28, 93–97. https://doi.org/10.1179/bac.2003.28.1.018.

Chase, C.A., Sinclair, T.R., Chellemi, D.O., Olson, S.M., Gilreath, J.P., Locascio, S.J., 1999. Heat-retentive films for increasing soil temperatures during solarization in a humid, cloudy environment. Hortscience 34, 1085–1089. https://doi.org/10.21273/HORTSCI.34.6.1085.

Chauhan, Y.S., Ghaffar, M.A., 2002. Solar heating of seeds - a low cost method to control bruchid (*Callosobruchus* Spp.) attack during storage of pigeonpea. J. Stored Prod. Res. 38, 87–91.

Chinwada, P., Giga, D.P., 1996. Sunning as a technique for disinfesting stored beans. Postharvest Biol. Technol. 9, 335–342. https://doi.org/10.1016/s0925-5214(96)00010-5.

Fawki, S., Abdel Fattah, H., Hussein, M., Ibrahim, M., Soliman, A., Salem, D., 2014. The use of solar energy and citrus peel powder to control cowpea beetle Callosobruchus maculatus (F.)(Coleoptera: Chrysomelidae), In: Arthur, F.H.K., R.; Chayaprasert, W.; Suthisut, D. (Eds), Proceedings of the 11th International Working Conference on Stored Product Protection, 24-28 November, 2014, Chiang Mai, Thailand, pp. 1022-1033. 10.14455/DOA.res.2014.168.

Fawki, S., Yousery, A., 2021. Dataset of Thermal Behaviour and Weather Data of Thermal Disinfestation of *Sitophilus oryzae* in Plastic Bags Using Solar Heating. Mendeley Data. https://doi.org/10.17632/j9c5mcmw3c.1. V1.

Fields, P.G., 1992. The control of stored-product insects and mites with extreme temperatures. J. Stored Prod. Res. 28, 89–118. https://doi.org/10.1016/0022-474X(92)90018-L.

Jian, F., Jayas, D.S., White, N.D.G., 2009. Temperature fluctuations and moisture migration in wheat stored for 15 months in a metal silo in Canada. J. Stored Prod. Res. 45, 82–90. https://doi.org/10.1016/j.jspr.2008.09.004.

Jian, F., Jayas, D.S., White, N.D.G., 2013. Specific heat, thermal diffusivity, and bulk density of genetically modified canola with high oil content at different moisture contents, temperatures, and storage times. Trans. ASABE 56, 1077–1083. https://doi.org/10.13031/trans.56.10067.

Joudi, K.A., Farhan, A.A., 2014. Greenhouse heating by solar air heaters on the roof. Renew. Energy 72, 406–414. https://doi.org/10.1016/j.renene.2014.07.025.

Kitch, L.W., Ntoukam, G., Shade, R.E., Wolfson, J.L., Murdock, L.L., 1992. A solar heater for disinfesting stored cowpeas on subsistence farms. J. Stored Prod. Res. 28, 261–267.

Kıyan, M., Bingöl, E., Melikoğlu, M., Albostan, A., 2013. Modelling and simulation of a hybrid solar heating system for greenhouse applications using Matlab/Simulink. Energy Convers. Manag. 72, 147–155. https://doi.org/10.1016/j.enconman.2012.09.036.

Kumar, D., Kalita, P., 2017. Reducing postharvest losses during storage of grain crops to strengthen food security in developing countries. Foods 6, 8. https://doi.org/10.3390/foods6010008.

Lale, N.E.S., Vidal, S., 2003. Simulation studies on the effects of solar heat on egg-laying, development and survival of *Callosobruchus maculatus* (F.) and *Callosobruchus subinnotatus* (Pic) in stored Bambara groundnut *Vigna subterranea* (L.) Verdcourt. J. Stored Prod. Res. 39, 447–458.

Lamont, W.J., 1996. What are the components of a plasticulture vegetable system? HortTechnology 6, 150–154. https://doi.org/10.21273/horttech.6.3.150.

Manandhar, A., Milindi, P., Shah, A., 2018. An overview of the post-harvest grain storage practices of smallholder farmers in developing countries. Agriculture 8, 57. https://doi.org/10.3390/agriculture8040057.

McGill, J., Prikhodko, D., Sterk, B., Talks, P., 2015. Egypt: Wheat Sector review. FAO Investment Centre. Country Highlights (FAO). FAO/EBRD, Rome, Italy no. 21.

Muhammad, A.A., Akhlaque, A., Tasneem, A., Muhammad, A., 2006. Use of solar radiation at village level for thermal disinfestation of wheat stored in galvanized steel bins. Pak. Entomol. 28, 57–64.

Murdock, L.L., Shade, R.E., 1991. Eradication of cowpea weevil (Coleoptera: Bruchidae) in cowpeas by solar heating. Am. Entomol. 37, 228–231. https://doi.org/10.1093/ae/37.4.228.

MWPS-22, 1980. Low Temperature and Solar Grain Drying Handbook Midwest Plan Service. Iowa Statw Univsersity, Ames, Iowa.

Ntoukam, G., Kitch, L.W., Shade, R.E., Murdock, L.L., 1997. A novel method for conserving cowpea germplasm and breeding stocks using solar disinfestation. J. Stored Prod. Res. 33, 175–179. https://doi.org/10.1016/S0022-474X(96)00042-2.

Okram, S., Hath, T.K., 2019. Biology of *Sitophilus oryzae* (L.) (Coleoptera: Curculionidae) on stored rice grains during different seasons in terai agro-ecology of West Bengal. Int. J. Curr. Microbiol. Appl. Sci. 8, 1955–1963. https://doi.org/10.20546/ijcmas.2019.804.229.

Rajendran, S., Sriranjini, V.-r., 2007. Use of fumigation for managing grain quality. Stewart Postharv. Rev. 3, 1–8. https://doi.org/10.2212/spr.2007.6.9.

Rita Devi, S., Thomas, A., Rebijith, K.B., Ramamurthy, V.V., 2017. Biology, morphology and molecular characterization of *Sitophilus oryzae* and *S. zeamais* (Coleoptera: Curculionidae). J. Stored Prod. Res. 73, 135–141. https://doi.org/10.1016/j.jspr.2017.08.004.

Sallam, M.N., 2000. INSECT DAMAGE Post-harvest Operations. Food and Agriculture Organization. http://www.fao.org/publications/card/en/c/8d4df8eb-6d58-4594-87a0-ebf8bc55cb54/. (Accessed 26 June 2020).

Singh, K., Agrawal, N.S., Girish, G.K., 1974. The oviposition and development of *Sitophilus oryzae* (L.) in different high-yielding varieties of wheat. J. Stored Prod. Res. 10, 105–111.

Smith, C.V., 1969. Meteorology and Grain Storage. World Meteorological Organization - WMO. No. 243).

Stapleton, J.J., 1996. Fumigation and solarization practice in plasticulture systems. HortTechnology 6, 189–192. https://doi.org/10.21273/horttech.6.3.189.

Strang, T., 2012. Studies in Pest Control for Cultural Property. Department of Conservation; Institutionen För Kulturvård. University of Gothenburg. Faculty of Science, Gothenburg, Sweden.

Strang, T., Kigawa, R., 2009. Combatting pests of cultural property. In: CCI Technical Bulletin. Canadian Conservation Institute, Department of Canadian Heritage, Ottawa, Canada.

Strang, T.J.K., 2014. The use of thermal control against insect pests of cultural property, In: Arthur, F.H.K., R.; Chayaprasert, W.; Suthisut, D. (Eds), The 11th International Working Conference on Stored Product Protection, 24-28 November, Chiang Mai, Thailand, pp. 690-723. 10.14455/DOA.res.2014.110.

Subramanyam, B., Flinn, P., Mahroof, R., 2002. Development and validation of a simple heat accumulation model for predicting mortality of *Tribolium castaneum* (Herbst) first instars exposed to elevated temperaturesCredland, P.F., Armitage, D.M., Bell, C. H., Cogan, P.M., Highley, E. (Eds.), In: Proceedings of the 8th International Working

Conference on Stored Product Protection, 22-26 July 2002, York, UK. CAB International, Wallingford, United Kingdom, 2003, pp. 369–374.
Taki, M., Rohani, A., Rahmati-Joneidabad, M., 2018. Solar thermal simulation and applications in greenhouse. Inform. Process. Agric. 5, 83–113. https://doi.org/10.1016/j.inpa.2017.10.003.
Tang, J., 2007. Heat Treatments for Postharvest Pest Control : Theory and Practice. CABI Pub., Wallingford, Oxfordshire, UK; Cambridge, MA.
Venkidusamy, M., Jagadeesan, R., Nayak, M.K., Subbarayalu, M., Subramaniam, C., Collins, P.J., 2017. Relative tolerance and expression of resistance to phosphine in life stages of the rusty grain beetle, *Cryptolestes ferrugineus*. J. Pest. Sci. 91, 277–286. https://doi.org/10.1007/s10340-017-0875-7.
Yan, R., Huang, Z., Zhu, H., Johnson, J.A., Wang, S., 2014. Thermal death kinetics of adult *Sitophilus oryzae* and effects of heating rate on thermotolerance. J. Stored Prod. Res. 59, 231–236. https://doi.org/10.1016/j.jspr.2014.03.006.
Zettler, J.L., Keever, D.W., 1994. Phosphine resistance in cigarette beetle (Coleoptera: Anobidae) associated with tobacco storage in the south eastern United States. J. Econ. Entomol. 87, 546–550. https://doi.org/10.1093/jee/87.3.546.
